# Understanding Agency in Woman’s Economic Empowerment Policy: an Analysis of Three Programs in India

**DOI:** 10.12688/gatesopenres.16377.1

**Published:** 2026-02-27

**Authors:** Shruti Ambast, Pallavi Khare, Vedavati Patwardhan, Lotus McDougal, Katherine Hay

**Affiliations:** 1 University of California San Diego, San Diego, California, USA

**Keywords:** gender, economic empowerment, agency, India

## Abstract

The concept of agency has gained prominence in development discourse over the past few decades, reflecting a shift away from top-down approaches and recognition of how individuals and communities shape how and whether programs work. This growing emphasis on agency, and calls for policies and programs that build or expand agency across all major international development funders and implementers, has brought increasing attention to what agency means, how it is shaped, and how to measure it. While research on agency at the intervention level has grown, there has been almost no published analysis of how this growing attention has found home in public policies.

In this study, we examine three economic empowerment schemes in India. We adapt an existing framework for empowerment to develop a policy analysis tool and apply it to a textual analysis of the selected schemes.

We find several key aspects of agency embedded in the reviewed schemes. All three schemes acknowledge existing gender gaps in access to basic resources and services, and make select resources available and accessible to women (factors which can potentially enhance agency). However, the schemes have less reference to control over resources and the conditions shaping that control. For example, interventions targeting intra-household dynamics and social norms around women’s work are generally absent. Likewise, while collective action and connections with other social structures are encouraged in the schemes, contextual and normative factors governing women’s ability to act for themselves in such structures are largely unmentioned.

The addition of measures targeted at women over time in the schemes suggests growing policy intent around gender equity and agency. Given this intent, we believe this type of policy analysis has promise in suggesting pathways for strengthening agency in the design of individual policies or schemes.

## Introduction

Gender scholars and activists have long argued that women’s agency is a critical component of empowerment, both an outcome in its own right, and a component of larger empowering processes that shape and modify other outcomes ( Kabeer, 1999; Ibrahim and Alkire, 2007). Agency is broadly understood as the ability to identify goals and act upon them. Increasingly, governments and funders have also stated that empowerment and agency are core to their funding and focus. The increasing focus on agency is rooted in growing recognition that improving access to resources and services may be inadequate in the absence of agency to seek out, use, or control them. Strengthening women’s agency has thus become an increasing part of discourse around gender equality ( World Bank, 2017; Raj, 2020).

This has led to an increase in research measuring or evaluating the impact of various interventions on agency. For example, a 2020 review that looked at 160 randomized controlled trials of interventions to strengthen agency found evidence on the positive impact of some interventions on multiple indicators of agency ( Chang et al., 2020). However, to date there has been very little work evaluating the extent to which agency is addressed in the design of public policy other than a few feminist and sociological studies examining how policies and institutional structures impact agency over time ( Mueller, 2015; Burgess and Campbell, 2015). While various gender analysis frameworks ( Longwe 1991; Kabeer and Subrahmanian, 1996; March et al., 1999) have been used at the policy level to evaluate gender equality indicators and budgets, they have not explicitly included or focused on constructs of women’s agency.

Given the importance of government policy and investment in shaping development outcomes, and the growing focus and attention to agency as a critical lever of development, we believe more analysis is needed to assess whether, and how, agency is integrated in government policies and programs.

In this study, we modify the EMERGE framework of empowerment which centers on agency, and apply it in a policy analysis ( Raj et al., 2024). Given India’s focus on women’s economic empowerment and the presence of large economic programs with gender dimensions, we test the framework in this context. Using textual analysis, we examine how three economic empowerment programs in India implicitly and explicitly integrate agency: Mahatma Gandhi National Rural Employment Guarantee Scheme (MGNREGS), National Rural Livelihoods Mission (NRLM), and Pradhan Mantri MUDRA Yojana or MUDRA. We believe the findings reflect how discourse around agency is shaping, and could strengthen, large scale policies and programs in the country.

### Women’s economic agency in india and selection of schemes for analysis

Indian policy level efforts are often operationalized into large ‘schemes’ and both the Union and state governments have a number of schemes in place that directly or indirectly address concerns of women’s economic empowerment and agency. Schemes cover social protection such as guaranteed wage employment; skill development; mobilizing women into Self-Help Groups (SHGs) for collective action; reducing women’s burden of unpaid care work; legal guarantees against discrimination; and others ( IWWAGE, 2020). There are over fifty schemes designated by the Union Government as ‘core schemes’ and six schemes designated as ‘core of the core schemes’ ( Government of India, 2024). These schemes are considered part of the national development agenda, and the latter have the first claim on available funds for development ( Government of India, 2016). Of these schemes, we selected two for analysis: Mahatma Gandhi National Rural Employment Guarantee Scheme (MGNREGS) and National Rural Livelihoods Mission (NRLM), owing to their large size and priority. In 2022-23, MGNREGS provided work to over 60 million households and NRLM mobilized over 90 million women into self-help groups or SHGs ( Ministry of Rural Development, 2023a). We added a third scheme, Pradhan Mantri MUDRA Yojana or MUDRA (Micro Units Development and Refinance Agency Limited), a relatively new initiative that eases access to credit and centers the individual beneficiary. Though much smaller, we hypothesized that given its launch five years after NRLM and ten years after MGNREGS, it might reflect changes in policy paradigms around agency over that period.

MGNREGS and NRLM were both introduced during a decade of rights-based movements and discourse. During this period, a basket of legal guarantees to social protection was introduced, including the Right to Information Act, 2005; MGNREGA, 2005; Forest Rights Act, 2009; Right to Education, 2009; and the National Food Security Act, 2013. These guarantees involved a significant commitment of public resources ( Kühner and Nakray, 2017; Tillin, 2021) and were positioned as an acknowledgement that the rural poor required assured access to basic services and social protection. In the following decade (2014-2024), many policies focused on expanding individual access to specific inputs or assets such as LPG cylinders, bank accounts, toilets, housing, and credit. The MUDRA scheme can be seen as reflecting this approach.

MGNREGS was enacted in 2005, with the objective of guaranteeing 100 days of work annually to rural households ( Ministry of Rural Development, 2023b). The scheme was also intended to create community infrastructure in rural areas. The scheme provides a safety net to vulnerable households, particularly in times of distress, mostly recently witnessed during the COVID-19 pandemic ( Afridi et al., 2022). Women’s participation in the scheme has been around fifty percent or more for several years, well above the earmarked minimum of 33 percent in the legislation ( Ministry of Rural Development, 2023c). The scheme has several provisions that cater to women’s needs (such as provision of crèches near work sites) and suggests a level of gender-responsiveness in design.

NRLM (since renamed Deendayal Antyodaya Yojana or DAY-NRLM) was launched in 2011 ( Ministry of Rural Development, 2015). NRLM’s mandate is to link rural poor households with sustainable livelihood opportunities. The scheme operates through forming ‘institutions of the poor’– which include Self-Help Groups (SHGs), SHG Federations, and livelihood collectives, and linking them to mainstream institutions like banks and government departments. The majority of SHGs are composed of women, and over time, many measures have been initiated aimed directly at improving gender outcomes in areas like livelihoods and access to finance, and addressing gender-based violence.

MUDRA was launched in 2015 to provide loans to small enterprises ( MUDRA Ltd., 2024). Three categories of loans are offered under the scheme: Shishu (loans up to Rs. 50,000); Kishor (loans between Rs. 50,000 – Rs. 5 lakh); Tarun (loans between Rs. 5 – 10 lakh). Women entrepreneurs are explicitly encouraged under the scheme to apply and MUDRA provides a reduced interest rate to banks and micro-finance institutions that offer loans to women. The scheme emphasizes self-employment as a path to poverty reduction.

### Using a conceptual framework to assess agency

Various frameworks and measures have been used to understand women’s agency generally and as an outcome or pathway in intervention research. For example, Richardson et al. (2019) measured agency among women in rural India using 40 indicators of agency across 4 domains (household decision making, freedom of movement, participation in the community, attitudes and perceptions). Chang et al. (2020) included four direct indicators of agency: power within (referring to beliefs and perceptions of self-worth, capabilities), household decision making, freedom of movement, and freedom from violence, and proposed a set of indirect indicators including timing of marriage and childbirth, contraception use, labor force participation, and political participation. However, neither of these frameworks have been used to evaluate policy.

Gender analysis frameworks more broadly have been applied to evaluate policy. For example, The Gender Roles Framework ( March et al., 1999) was used to evaluate India’s forest policy ( Tyagi and Das, 2018). The Social Relations Framework ( Kabeer and Subrahmanian, 1996) proposes a classification for gender policies, as gender-blind, gender-aware, and gender-redistributive and has influenced many analyses. The Women’s Empowerment Framework ( Longwe, 1991) was adapted to analyze Indian policies on women’s economic empowerment in 2020, with each policy/scheme categorized as ‘negative’, ‘neutral’, or ‘positive’ against five levels of empowerment ( Kumar et al., 2020). However, neither of these frameworks have an explicit inclusion of agency and its constructs.

Finally, few policy analyses address agency as an outcome, but do not draw on a framework built for policy analysis; for example, Mueller (2015) analyzes reservations for women in local governance in India, showing how norms and local discourse can impede women’s political agency.

In this context, we believe a framework and tool for assessing agency in policies is a useful contribution.

### Adapting the EMERGE framework for policy analysis

**Figure 1.**
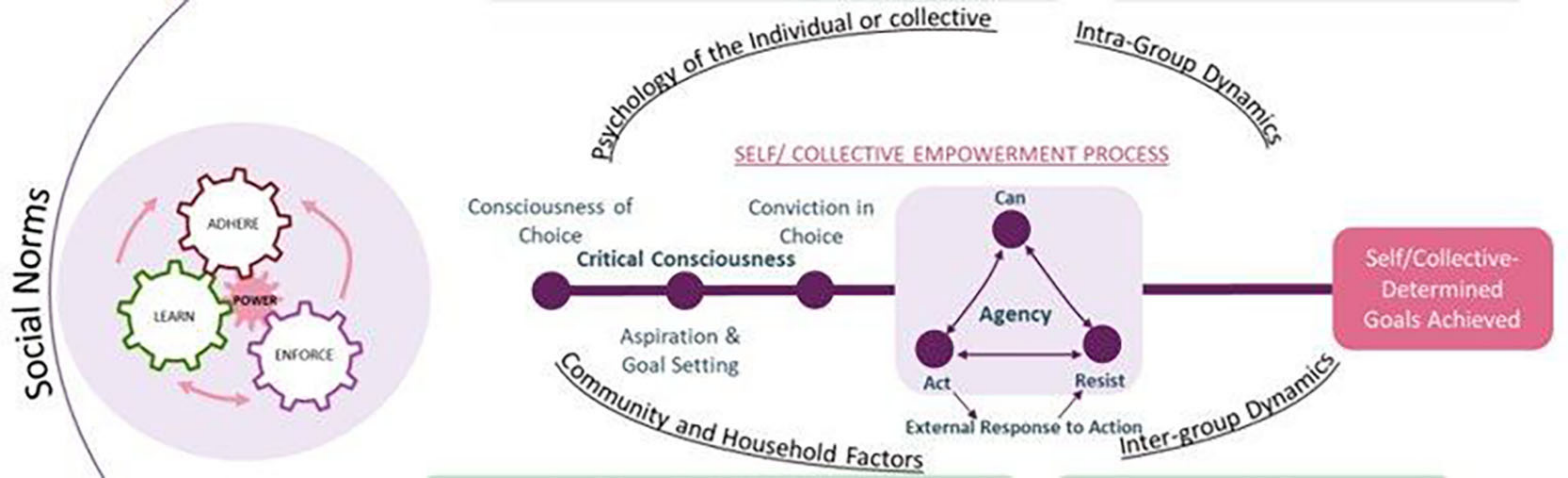
Conceptualization of the empowerment process and locating its inputs. Note: This figure has been adapted with permission from Raj et al. (2024). ‘The EMERGE framework to measure empowerment for health and development.’ *Soc Sci Med.* 2024/06/01/2024;351:116879. Retrieved January 10, 2025, from https://doi.org/10.1016/j.socscimed.2024.116879.

As agency is complex and dynamic, evaluating its integration in policy requires a conceptual framework effectively describing agency. The EMERGE framework (Evidence-Based Measures of Empowerment for Research on Gender Equality), developed by researchers at the Center on Gender Equity and Health (GEH) aims to fill this gap ( Raj et al., 2024). The framework draws on theories of empowerment from multiple disciplines, including economics, psychology, sociology, and political science. It describes a non-linear process of empowerment for individuals and collectives that begins with critical consciousness, is followed by aspiration and goal-setting, acting upon the goals decided, and culminating in the achievement of these self-determined goals ( Figure 1).

**Agency** in the EMERGE framework is understood through the interrelated elements of ‘Can-Act-Resist.’ *Can* refers to efficacy or capacity to act, at the level of the self or collective. Efficacy may be perceived or actual in the form of control over assets and resources. *Act* refers to the actions of individuals or collectives in line with their goals, and may include giving voice or communicating one’s goals, decision-making about issues affecting one’s goals, or simply engaging in direct actions to achieve one’s goals. These actions involve direct or covert communication with people in positions of power. *Resist* refers to what actors do in response to negative external responses or the backlash to *Act.* It involves direct defiance of power structures, or engaging the power structures through bargains or compromises.

**Social norms** are also a key part of the EMERGE framework. Norms are informal, unwritten rules that govern behaviour, and are often the reason that inequalities persist. Entities that influence how norms are learned, adhered to, and enforced include: power-holders (such as politicians, religious leaders, patriarchs), and reference groups (such as peers, family, community). Finally, the framework includes **two kinds of inputs** that act upon the empowerment process: internal attributes and external context. **Internal attributes** are psychological and emotional traits that may facilitate or inhibit empowerment for individuals or collectives. For instance, knowledge of choice and psychological resilience may help one resist power structures. **External context** includes the larger sociopolitical environment, where policies and social and political movements have an important role to play. It includes community and household dynamics that determine how assets and resources are distributed.

The EMERGE framework was developed to strengthen the measurement and monitoring of gender indicators, and not as a policy analysis tool. However, the conceptualization of empowerment within the framework has several intersections with policy.

Broadly speaking, policy refers to the larger policy regime or environment, institutions, laws, regulations, public infrastructure and services, and welfare schemes. Policies and programs can create supportive infrastructure, redistribute assets and resources, facilitate access to services, and institute systems of incentives and sanctions; all of which have a bearing on women’s agency. Within the EMERGE framework, policy forms part of the external context of an individual or a group – that can facilitate or impede empowerment. Targeted policy interventions can be used to affect other inputs to the empowerment process, such as the knowledge of options, attitudes and beliefs, social networks, and social support.

For our analysis, we draw upon the component constructs of agency and external context as articulated in the framework. Agency-related constructs are possessed or exercised by an individual or a group, but public policy is able to influence them. For example, public investment in safe public transport can potentially shape women’s goals, their actions towards those goals, or their achievement of those goals. Policy actions and investment can influence how agency is exercised at an individual or collective level, by enabling access to resources or creating platforms for action. Agency, in turn, can lead to the introduction of new policies or changes in existing ones, through mobilization and collective action.

### Creating a Policy Analysis Instrument

To create our policy analysis instrument, we mapped a set of questions against three component constructs of agency to ascertain how schemes integrate these constructs. We divided the first two constructs (‘can’ and ‘act’) into constituent domains (total of five) to express different ways in which the construct is operationalized to link to the role of policy. ‘Can’ is broken down into two domains: (i) self-efficacy to control key life resources, and (ii) self-efficacy to obtain and use services. Both domains speak to the capacity of an individual or collective to take action towards their goals. It is assumed that access to and control over basic resources, combined with critical consciousness about their importance, is critical to self-efficacy, even if these resources are delivered by the government. ‘Act’ is broken down into three domains covering decision making relating to goals, platforms for collective action, and supportive external context.

Against each construct of agency (and their constituent domains), we identified ‘transformative’ questions of whether the scheme speaks to women’s agency, and practical questions that probe specific provisions and activities within schemes that speak to the transformative questions. A total of fifteen practical questions were identified. An example from the instrument is shown below.

**Table:** 

Construct of agency	Domain	Transformative question	Practical question
Can – Actors’ capacity to engage in action	Self-efficacy to obtain and use services	Does the scheme speak to limitations on women’s access and use of services and seek to expand both in a way that enhances their agency?	Proximity of accessing service: Does the scheme design include coverage to the last mile (village level)?
Act – Actions aligned to choices and goals	Platforms for collective action and social support	Does the scheme speak to supportive intra- and inter-group dynamics that can facilitate women’s action?	Group formations: Does the scheme design include interventions to form groups amongst women – e.g. self-help groups?

Each question was then answered with reference to the scheme as a whole, and any relevant specific scheme components. The response to each question was coded to indicate: (1) whether the scheme explicitly targets women/girls OR is a ‘general’ intervention that also benefits women/girls; and (2) whether components are mandatory (either backed by legislation or mandated by policy direction) or recommended. Responses clustered within the agency domains were then analyzed to explore the inclusion or absence of agency considerations across domains and overall.

*Limitations*

We confined our source material to public documents laying out the schemes’ design and content, as our objective was to examine the stated or written intent of schemes. Our analysis does not extend to the outcomes or impacts of these policies (whether this intent was realized) or the process of implementation and whether policies rolled out as intended and planned. The reference material for the schemes includes guidelines and directions issued by the Union Government of India. States have expanded existing scheme provisions or added activities and measures of their own; these were not considered for the present analysis.

### Findings

The findings on how agency is included in the different schemes are grouped by the three EMERGE framework components of Can-Act-Resist.

### CAN: Supporting the capacity to act

**‘CAN’** refers to efficacy or the capacity to act, at the level of the self or collective. It may be perceived efficacy, or actual efficacy in the form of control over assets and resources. We divide the construct into two domains: control over key life resources and assets, and obtaining and using services.

In any context with gender inequities in resource distribution, delivery of resources and services by government schemes can be a route towards greater self-efficacy for women. We see this distribution of services across all three schemes:

MGNREGS (Mahatma Gandhi National Rural Employment Guarantee Scheme) guarantees 100 days of wage employment for rural households, with 33% of benefits earmarked for women, revealing an explicit gender focus. The scheme:

• Provides entitlements such as work near home, travel allowance, worksite facilities, and unemployment allowance. Access to these entitlements can be seen as a critical input in the agentic process; that is, it secures women against unemployment and economic distress, and so improves their ability to act towards self-determined goals.
• Offers skill development opportunities that can serve as routes for women to develop greater self-efficacy.
• Has provisions informed by gendered constraints on access such as the entitlement for work to be provided near one’s home and the provision for crèches at worksites with a minimum number of children accompanying their mothers. These address two key existing limitations on women’s access to paid work - mobility restrictions and the disproportionate burden of unpaid care work.
• Enhances access to services through multiple registration platforms and modes.

Women availing these entitlements may stand to gain in overall self-efficacy to the extent that this corrects for gaps in access to basic resources and services. But their capacity to act may yet be hindered by other factors not addressed by the scheme: intra-household dynamics that determine decisions to work and control over resources; and social norms prevalent in the community and society at large about women doing paid work.

NRLM (National Rural Livelihoods Mission) facilitates rural households’ access to SHGs and local networks, and to a range of other resources that can be considered important for self-efficacy, like skills training, financial literacy, infrastructure for livelihood support, and capital. The program’s intent is that access will provide pathways out of poverty and ultimately lead to self-sustaining improvements in employment and economic empowerment ( Ministry of Rural Development, 2015). SHGs are primarily composed of women, so many of the scheme’s provisions have an explicit gender focus ( Ministry of Finance, 2023a). The scheme:

• Acknowledges the need to build women’s capacity to act at a collective level; the underlying assumption may be that women in SHGs can exercise a degree of control over resources that they cannot do individually.
• Forms and expands the capacities of existing SHGs (and links them to resources) thus acknowledging resource deficits that women experience even in social structures that are considered empowering.
• Provides resources like skills training, financial literacy, infrastructure for livelihood support, and capital.
• Promotes last-mile delivery of public services and earmarks benefits for vulnerable communities, ear marking benefits and expanding access to services for individuals and groups who may not otherwise have access - a valuable component of self-efficacy.
• Recognizes the need for collective action to enhance women’s capacity to act.

MUDRA (Micro Units Development and Refinance Agency): The MUDRA scheme aims at easing access to credit by refinancing small banking institutions and microfinance institutions (MFIs) that serve small businesses and micro entrepreneurs. The loans provided under the scheme do not require collateral, which implicitly benefits women as they are less likely to have assets for collateral ( Sarkar and Kumar, 2022). The annual reports of the scheme, as well as multiple press releases of the government, note the large proportion of women beneficiaries (68% of loan accounts belonged to women in 2021-22) as a successful outcome ( Ministry of Finance, 2023b). The scheme:

• Speaks to existing barriers on women’s access to finance, and provides access to a resource that can enhance self-efficacy.
• Aims to bring more women in contact with formal financial institutions which could improve consciousness of choice around services.

The planned future strategy for MUDRA ( MUDRA Ltd., 2024) mentions several interventions including building financial and business literacy, setting up credit counseling centers, developing knowledge of financial products, and setting up incubators for grassroots innovations, etc. The scheme does not speak to social norms or the intra-household dynamics that may determine whether a woman chooses to avail the loan, how much control she has over the amount, or how she uses it.

All three programs address access to resources and services, to varying degrees and through different approaches. MGNREGS focuses on providing guaranteed employment and addressing specific barriers to women’s participation in the workforce. NRLM takes a more comprehensive approach, emphasizing collective action through SHGs and providing a range of resources to build women’s capacities. MUDRA primarily addresses financial efficacy by improving access to credit.

While all three programs contribute to enhancing women’s capacity to act through the provision of services, they do not fully address factors affecting women’s ability to control these resources. Intra-household dynamics, social norms, and broader societal constraints on women’s economic participation are not directly tackled. NRLM, in its provision of multiple interlinked interventions, appears to have the most comprehensive approach to building efficacy, recognizing that access to one kind of resource (such as credit) may not be meaningful for economic empowerment without access to others (networks, infrastructure, information). MGNREGS provides a crucial safety net that can enhance economic efficacy, while MUDRA focuses narrowly on financial access. Overall, these programs represent significant efforts to enhance women’s efficacy or capacity to act, with room for more holistic approaches that address the conditions in which women control resources and make decisions about their use.

### ACT: Supporting action by individuals or collectives

The ‘Act’ domain of agency comprises the **actions taken by individuals or collectives that are aligned to their goals,** with or without the knowledge of those in positions of power. The role of public policies here is not straightforward, as the state is an external power structure whose goals and choices may be at odds with those of the people. However, the state may create and provide access to platforms where individuals (and collectives) can exercise choice and make decisions in line with their goals. It may provide services that are directly aligned to specific goals (like education or employment). Finally, policies can create a supportive external context that is responsive to people’s (in this case, women’s) actions towards their goals by collecting and publishing aggregate data on important indicators and involving beneficiaries in policy design.

Work under ***MGNREGS*** may not be an exercise of agency, but a fallback in the absence of better opportunities ( Kumar, 2022). Within the ambit of the scheme, however, there are ways in which women’s actions are supported.

• MGNREGS work schedules are approved by the Gram Sabha, where women’s representation is already mandated, thus creating an avenue for women to influence the public works undertaken.
• Vigilance and Monitoring Committees (VMCs) are required to be set with adequate representation from Scheduled Castes and Scheduled Tribe households, half of whom are required to be women.
• SHGs are encouraged to be implementing agencies in general, and specifically for certain projects like watersheds, thus explicitly ensuring women’s participation.
• New interventions added under the scheme do suggest some degree of gender-responsiveness, like promoting women supervisors (Mahila Mates) for work sites.
• Multiple monitoring measures exist, with data on scheme implementation being collected through an MIS and published on the website, a provision, ‘Rozgar Diwas’ for local monitoring and filing of grievances, and functionaries required to verify different activities, ensure inclusion of vulnerable groups and take remedial measures. This suggests a supportive external context where certain outcomes are tracked and can potentially provide a feedback loop to inform changes in policy design or implementation.

These are routes for women to individually and collectively make decisions about community development and monitor the effectiveness of the scheme. None of these measures, however, speak to barriers that may prevent women from acting in their own (individual or collective) interests, within such structures, such as elite capture or power imbalances in local institutions. The measures largely build on existing structures of decision making and do not speak to norms or social hierarchies inhibiting women’s agency within those structures.

***NRLM*** supports a variety of direct actions by women: to save, enhance skills and knowledge, access networks, and pursue paid work/entrepreneurship, though the livelihood opportunities may not be aligned to women’s own aspirations and goals. Again, let’s look at how the program creates and provides access to platforms, services, and monitoring where individuals and groups can exercise choice and make decisions in line with their goals.

• The emphasis on SHGs as a platform is a recognition of the constraints upon individual women to take action. Membership of SHGs and their federations that are supported under the scheme may help strengthen intra- and inter-group dynamics, making it easier for women to take action.
• The scheme promotes engagement of SHGs with Panchayati Raj Institutions, NGOs, civil society organizations, livelihood collectives, Anganwadis, and schools; this can help SHGs build useful alliances and gain positive recognition from those in positions of power. In 2018, an intervention was introduced to train SHGs to participate in planning for poverty reduction and development in villages ( Ministry of Rural Development, 2020).
• Counselling for skill development is provided under the scheme, which may support decision making. In 2019, the National Rural Economic Transformation Project (NRETP) was launched in partnership with the World Bank to support enterprise development for women ( Ministry of Finance, 2019).
• There is an institutional architecture, including village-level committees and gender forums, to monitor and report progress on scheme activities. Monitoring mechanisms for NRLM operate at national, state, district, and local levels. States are also free to come up with their own mechanisms for greater accountability. Thus there are ways for women to potentially have a say in how the scheme is implemented.
• In 2016, gender mainstreaming was introduced in NRLM through a variety of mechanisms, including sensitization of staff and introduction of institutional mechanisms where women could voice and resolve issues ( Ministry of Rural Development, 2021).

It is not clear how effective this kind of facilitation is in guiding women towards remunerative livelihoods in line with their own aspirations. Access may be provided only to specific skills or markets deemed suitable within the scheme’s design.

***MUDRA*** provides a specific financial service that may be availed by women at existing platforms (banks and other lending institutions); women’s choice to avail loans is made easier as institutions are incentivized to provide collateral-free loans to women. Women beneficiaries can choose among three categories of loans of varying size. Most women under the scheme opt for the smallest category (‘Shishu’ – up to INR 50,000) ( MUDRA Annual Report, 2022-23), suggesting that the size of their entrepreneurial activities is small, and may be hindered by other factors (such as lack of financial literacy, lower access to business networks, or restrictive norms).

MUDRA’s scope is limited, especially in comparison to the other two schemes discussed here; it does not have measures to support decision making by women, counseling, or platforms to influence program design. The service is simply made available, the indicated assumption being that women are otherwise free to leverage it for greater well-being. Future synergies are envisioned with NRLM, along with strategies to formalize grassroots institutions and incubate grassroots innovations, which may have a supportive impact upon women entrepreneurs; but these have not yet been actioned. MUDRA also reports gender-disaggregated beneficiary data across loan categories. However, this data only captures specific outcomes and not the process, or experience of quality of services, or aspirations, which may be more relevant to women’s ability to act upon their goals.

The three schemes demonstrate varying approaches to supporting women’s agency within the ‘act’ domain. MGNREGS and NRLM provide more comprehensive platforms for women’s participation in decision making and implementation, recognizing the importance of collective action. They also show evidence of evolving to be more gender-responsive over time. MUDRA, while providing financial choices, has a more limited scope in terms of supporting women’s agency beyond access to credit. All three schemes contribute to a supportive policy environment through data collection and reporting, though NRLM may have the most robust mechanisms for using this data to inform policy changes. However, across all schemes, there remains a question of whether the actions supported are truly aligned with women’s self-determined goals or are more focused on efficient service delivery. Additionally, the schemes generally do not address deeper societal norms and power dynamics that may limit women’s ability to act freely within these frameworks.

### RESIST: Supporting resistance to backlash

In the EMERGE framework when individuals or collectives face backlash for their actions from those who hold power over them, their continued action is referred to as resistance. Resistance may involve going against power structures, or bargaining/negotiating with them. Public policies can provide platforms for raising grievances and other accountability mechanisms, compensation for violations of guaranteed entitlements, sensitization for those in positions of authority, or any other measures that make it easier for women to negotiate with power holders. Such measures of accountability may have the effect of enhancing women’s conviction over choices, knowing that remedies and consequences exist, and can strengthen resilience. Strategies used by non-profits to mitigate backlash ( Pawar and Prabhughate, 2019) may be instructive for the design of government schemes and programs and include: engaging all important community stakeholders; having champions/role models; sensitizing men and boys; and having safety protocols in place, in anticipation of backlash. We looked for these components across the three programs.

***MGNREGS*** has provisions for grievance redress and social audits. Any worker or citizen can lodge a complaint under the grievance redress mechanism, which must then be resolved within a statutory time frame. Gram Sabhas are legally mandated to carry out social audits for the scheme, and social audit units are directed to include women from SHGs as resource persons. This suggests some recognition of the need to specifically engage women in accountability mechanisms. Vigilance cells at the state and district level, mandatory proactive disclosure of records, and notified principles of transparency are other measures that speak to the need for accountability in public provisioning. However, there are no interventions that explicitly address possible backlash against women’s participation, such as gender sensitization or counseling.

***NRLM***, on the other hand, has introduced provisions for gender mainstreaming that speak, in some measure, to the ‘resist’ construct. Besides a community-led grievance management system, Gender Justice Centers have been introduced - community-managed platforms that hear and resolve instances of gender-based violence; and gender sensitization sessions are held for program staff ( IWWAGE, 2022). This points to recognition of restrictive norms that may impede women from acting upon their goals, even under schemes that target their welfare.

***MUDRA***, notably, does not have a scheme-specific grievance redress mechanism. The scheme website states that grievances related to non-sanction of loans may be reported to higher authorities within the lending institutions.

While formal grievance redress mechanisms found in NRLM and MGNREGA may guard against visible violations of scheme guarantees and provisions, women may face backlash within their households and communities that deter them from fully availing these benefits or acting upon newly acquired resources. Only NRLM seems to consider this in some capacities.

## Discussion

This analysis examined three major Indian government schemes—MGNREGS, NRLM, and MUDRA—through the lens of the EMERGE framework to understand how they integrate and support women’s agency. The framework’s three components—Can, Act, and Resist—were used to evaluate the schemes’ intent around enhancing women’s capacity to act, supporting their actions, and addressing potential backlash.

All three schemes can be seen as contributing to women’s agency by providing access to resources and services, albeit through different approaches. MGNREGS focuses on guaranteed employment and related guarantees that address specific barriers to women’s work. NRLM takes a more comprehensive approach, emphasizing collective action through Self-Help Groups (SHGs) and providing a range of resources to build women’s capacities. MUDRA primarily addresses financial efficacy by improving access to credit for women entrepreneurs. Access to resources and services can enhance women’s self-efficacy (‘Can’), by equipping them to participate in the economic, social, and public spheres, and take actions aligned to their goals. The question of freedom to seek out resources and to have control over them, however, is not fully articulated or addressed in the design of the schemes.

While these programs enhance women’s capacity to act, they do not fully address factors affecting women’s ability to control these resources, such as intra-household dynamics and broader societal constraints. NRLM does acknowledge the multifaceted nature of empowerment, reflected in the emphasis on a variety of resources like skills, networks, capital, and financial literacy.

In terms of supporting women’s actions (‘Act’), MGNREGS and NRLM provide platforms for women’s participation in decision making and implementation, recognizing the importance of collective action. Women’s participation in the community, decision making in local development processes, and economic action through collectives (SHGs and their federations) is supported under both schemes. While action towards self-determined goals may be facilitated by these mechanisms, there is no explicit support to guard against tokenistic representation and elite capture by local power holders, which are documented phenomena in these settings ( Buch, 2016). The emphasis on collective action embodies an understanding that women may be better able to work towards certain goals in groups than as individuals. While some interventions encourage and even mandate women’s participation, are women free to choose to participate? For instance, SHG members being directed to monitor MGNREGS work sites, or being provided training in specific livelihood skills, may be more oriented to efficient service delivery than in alignment with their own goals. MUDRA, while providing financial choices, has a more limited scope in supporting women’s agency beyond access to credit. All three schemes contribute to a supportive policy environment through data collection and reporting; but data is reported mainly on select outcomes and not quality or experience of services. Both NRLM and MGNREGS have seen some gender-responsive policy changes over time, suggesting a supportive external context.

Regarding resistance to backlash, MGNREGS and NRLM have provisions for grievance redress and social audits, with NRLM introducing additional gender mainstreaming measures. MUDRA, however, lacks a scheme-specific grievance redress mechanism, potentially limiting its ability to address backlash against women’s participation.

Overall, these schemes represent significant efforts to enhance women’s agency, while still having room to expand approaches that address deeper societal norms and power dynamics.

### Reflections on using the EMERGE framework for policy evaluation

Applying the EMERGE framework to analyze MGNREGS, NRLM, and MUDRA has provided insight into both the policies themselves and the framework’s utility for policy evaluation.

We identified several strengths of the framework. The Can-Act-Resist structure offers a holistic view of women’s agency, allowing for a nuanced analysis of how policies support different aspects of empowerment. The framework was particularly effective at identifying components of existing policy that aligned with the framework, while still suggesting gaps where policies did not speak to or address deeper societal norms and power dynamics that limit women’s agency. The framework offers a systematic approach to comparison across different schemes, revealing their relative strengths and weaknesses in supporting women’s agency. Finally, the framework and our approach to using it (taking original policy documents and additions over time), enabled us to track how policies have evolved over time to become more gender-responsive, as seen in the case of NRLM’s gender mainstreaming initiatives.

The complexity of agency and context-dependent nature of women’s agency, was challenging to capture fully in textual policy analysis. This is partially to do with the lack of alignment of policy goals and agency: despite the broader policy discourse often speaking to empowerment and agency, predetermined objectives that can make these schemes effective and scaleable are often at odds with the framework’s focus on self-determined goals. Likewise the construct of resistance, which could include speaking out against government or other power brokers is arguably neither a focus nor desire of mainstream government programs and policies, but rather supported by government through legal entitlements. The exercise surfaced the complexities involved in translating theoretical constructs of agency into practical policy measures.

However, by examining different aspects of agency - capacity, action, and resistance – we believe the adaptation of the framework for policy analysis is useful for policy and program designers and evaluators who want to assess or consider how these schemes can be further aligned with women’s agency. We believe its continued use and adaption could support more nuanced, gender-responsive policy design and evaluation, potentially enhancing the effectiveness of women’s empowerment initiatives.

## Conclusions

A review of the literature suggests that agency matters for empowerment and gender equality and is a growing part of development discourse including around policy. However, while there are different frameworks that define and measure agency, none have been adapted to assess policy. This analysis is a first attempt to adapt an existing framework and use it to assess whether and how policies consider constructs of agency.

In applying the EMERGE framework to the three schemes, we observed the clearest focus on agency in the self-efficacy to own and control resources and assets. The success and size of the Indian government commitment to this exercise is well documented elsewhere and as clearly evidenced in the review. Conversely, the areas of intra-household decision making and social norms relating to women’s decision making components of self-efficacy were largely unacknowledged in scheme documentation. The existence and participation of women in structure and decision-making bodies was also extensively detailed in the schemes, while the dynamics governing women’s ability to act in collective settings was unacknowledged. Finally, the component of resistance was addressed to some extent through mechanisms of grievance redress, but this is a limited slice of what the concept embodies and arguably, not a goal of mainstream government programs.

Despite the limitations discussed, we believe this type of policy analysis has promise in suggesting pathways for strengthening agency in the design of individual policies or schemes, and in analyzing the construct of agency within and across changing policy paradigms over time.

## Data Availability

The data/information has been accessed from policy guidelines and literature available in the public domain; for these complete references have been provided, including links to the webpages wherever applicable. For requesting access to data, one may reach the authors at shrutiambast@gmail.com.
